# Addressing unmet clinical needs in treatment-resistant breast cancer: expanding the diagnostic–prognostic landscape through next-generation inflammatory signatures

**DOI:** 10.3389/or.2026.1918787

**Published:** 2026-10-16

**Authors:** Emmanuel Ifeanyi Obeagu

**Affiliations:** 1 Department of Biomedical and Laboratory Science, Division of Haematology, Africa University, Mutare, Zimbabwe; 2 Department of Molecular Medicine and Haematology, Faculty of Health Sciences, University of the Witwatersrand, Johannesburg, South Africa

**Keywords:** cytokine signatures, deep learning, inflammatory biomarkers, machine learning, prognostic indices, treatment-resistant breast cancer, tumor microenvironment

## Abstract

Treatment-resistant breast cancer remains a major clinical challenge because conventional clinicopathological and molecular biomarkers incompletely capture the dynamic interactions between tumour cells, immunity, inflammation, and the systemic host response. Next-generation inflammatory signatures offer an opportunity to expand the diagnostic–prognostic landscape by integrating cellular, molecular, immune, and thromboinflammatory indicators with established tumour characteristics. This narrative review critically examines emerging evidence for neutrophil-to-lymphocyte ratio, platelet-to-lymphocyte ratio, systemic immune-inflammation index, systemic inflammation response index, C-reactive protein, cytokines, tumour-associated macrophages, myeloid-derived suppressor cells, neutrophil extracellular traps, coagulation biomarkers, and liquid-biopsy-derived measures in treatment-resistant breast cancer. Particular attention is given to sources of heterogeneity, including population characteristics, tumour subtype, treatment exposure, comorbid inflammation, sampling time, specimen processing, analytical platforms, and inconsistent biomarker thresholds. The review further evaluates the transition from biomarker discovery to clinical decision support, highlighting the need for prospective multicentre validation, standardised analytical procedures, external validation of computational models, and integration with molecular subtyping, circulating tumour DNA, imaging, and immune profiling. Rather than interpreting inflammation as a static prognostic variable, longitudinal inflammatory trajectories may provide earlier signals of biological treatment resistance and complement conventional measures of disease progression. Future research should establish clinically actionable inflammatory combinations and evaluate biomarker-guided anti-inflammatory strategies alongside standard therapies. Ultimately, integrating inflammatory phenotypes with tumour molecular characteristics may facilitate earlier recognition of resistance and more precise, dynamic, and equitable breast cancer management.

## 1 Introduction

Breast cancer remains a biologically heterogeneous disease and a leading cause of cancer-related mortality among women worldwide. Despite substantial advances in screening, molecular classification, and targeted therapeutics, the emergence of treatment resistance continues to undermine long-term clinical outcomes. Resistance may be intrinsic or acquired and spans all major therapeutic modalities, including endocrine therapy, cytotoxic chemotherapy, HER2-targeted agents, and, more recently, immunotherapy. While genomic alterations and clonal evolution have traditionally dominated explanations of resistance, it is now clear that these tumor-intrinsic mechanisms operate within, and are profoundly shaped by, a dynamically regulated tumor microenvironment (TME) in which inflammation is a central organizing principle (1–3). Cancer-related inflammation is no longer viewed as a passive bystander but rather as an enabling hallmark of tumor progression. In breast cancer, persistent inflammatory signaling orchestrates a complex interplay between malignant cells, stromal components, and infiltrating immune populations (4, 5). Cytokines, chemokines, and growth factors—including interleukin-6 (IL-6), interleukin-8 (IL-8), tumor necrosis factor-alpha (TNF-α), and transforming growth factor-beta (TGF-β)—drive key oncogenic processes such as epithelial–mesenchymal transition (EMT), angiogenesis, immune evasion, and the maintenance of cancer stem–like cells. These processes collectively foster a permissive niche for therapeutic escape. Importantly, inflammatory signaling pathways such as NF-κB, JAK/STAT, and inflammasome activation establish self-reinforcing feedback loops that sustain tumor survival under pharmacologic pressure, thereby linking inflammation directly to treatment refractoriness (6, 7, 53).

Conventional biomarkers used in breast cancer management—such as hormone receptor status, HER2 expression, and proliferation indices—have significantly improved patient stratification but provide limited insight into the dynamic and systemic components of disease progression. Similarly, traditional inflammatory markers like C-reactive protein (CRP) lack specificity and fail to capture the spatial and temporal complexity of tumor-associated inflammation. This gap has catalyzed the development of next-generation inflammatory signatures, which integrate multidimensional data derived from tumor tissue, peripheral blood, and increasingly, non-invasive platforms such as liquid biopsy. These signatures move beyond single-analyte measurements to encompass coordinated networks of cytokines and chemokines, immune cell composition and functional states, and composite indices reflecting systemic inflammatory balance (8, 9, 56). Technological advances have been pivotal in enabling this shift. High-throughput transcriptomics, proteomics, and metabolomics now permit comprehensive profiling of inflammation-related pathways at unprecedented resolution. Single-cell sequencing and spatial transcriptomics further delineate the heterogeneity and spatial organization of immune infiltrates within the TME, revealing distinct cellular ecosystems associated with therapeutic response or resistance (10–12). Concurrently, computational approaches, including machine learning algorithms, facilitate the integration of complex datasets into clinically actionable predictive models. These developments have accelerated the identification of robust inflammatory signatures with diagnostic and prognostic relevance across diverse breast cancer subtypes, including triple-negative and HER2-positive disease, where treatment resistance is particularly problematic (13, 14).

From a clinical perspective, the appeal of inflammatory signatures lies in their dual capacity to reflect both tumor biology and host response. As dynamic biomarkers, they offer the potential for real-time monitoring of disease trajectory, early detection of resistance, and adaptive treatment stratification. For instance, shifts in cytokine profiles or immune cell ratios during therapy may precede radiographic progression, providing a critical window for intervention. Moreover, inflammatory signatures may inform therapeutic targeting, identifying patients likely to benefit from immunomodulatory or anti-inflammatory strategies in combination with standard regimens (15). This narrative review examines the emerging landscape of next-generation inflammatory signatures in treatment-resistant breast cancer. It synthesizes current understanding of the mechanistic links between inflammation and therapeutic failure, evaluates the evolving repertoire of inflammatory biomarkers, and explores their diagnostic and prognostic applications. By situating these advances within a translational framework, the review highlights the potential of inflammation-informed precision oncology to redefine clinical management in refractory.

Artificial intelligence (AI) is rapidly transforming the clinical management of non-small cell lung cancer (NSCLC), with applications spanning screening, medical imaging, radiomics, histopathological diagnosis, molecular profiling, treatment selection, immunotherapy response prediction, surgical planning, prognosis, and disease monitoring. Machine learning (ML), deep learning (DL), radiomics, pathomics, and large language models (LLMs) are increasingly being investigated as tools for extracting clinically meaningful information from complex and multidimensional datasets. Despite this rapid expansion, translation of these technologies into routine NSCLC care remains limited. Previous reviews have largely focused on individual AI applications, such as radiological diagnosis, radiomics, pathology, or prediction of treatment response. However, an important knowledge gap remains regarding how these rapidly developing approaches can be considered collectively across the entire NSCLC clinical pathway, particularly in relation to diagnostic accuracy, prognostic stratification, treatment selection, immunotherapy response, surgical decision-making, recurrence prediction, and longitudinal disease monitoring. Furthermore, many existing discussions place greater emphasis on algorithmic performance than on the practical barriers that determine whether AI models can be reliably generalised across institutions and integrated into clinical practice. This review addresses this gap by providing an integrated and clinically oriented assessment of AI applications across the NSCLC continuum, while simultaneously examining the challenges of data heterogeneity, imaging and pathological standardisation, feature reproducibility, multimodal data integration, missing data, interpretability, external validation, and prospective multicentre evaluation. Particular attention is given to the emerging convergence of radiomics, pathomics, molecular profiling, clinical variables, and advanced computational approaches, including ML, DL, and LLMs. Rather than viewing AI as a collection of isolated diagnostic technologies, we examine its potential as an interconnected decision-support framework capable of supporting precision oncology from early detection through treatment and long-term surveillance.

## 2 Methods

This narrative review was conducted to critically synthesize and interpret current evidence on next-generation inflammatory signatures in treatment-resistant breast cancer, with a focus on their diagnostic, prognostic, and translational relevance. The approach was designed to integrate mechanistic insights from molecular oncology, tumor immunology, and systems biology with emerging clinical and biomarker-based evidence. A structured literature search strategy was employed to identify relevant peer-reviewed studies published in indexed databases, including PubMed, Scopus, Web of Science, and Google Scholar. The search combined controlled vocabulary and free-text terms related to “breast cancer,” “treatment resistance,” “inflammation,” “cytokines,” “immune biomarkers,” “tumor microenvironment,” “liquid biopsy,” and “multi-omics signatures.” Additional search expansion was performed using reference tracking of key articles and reviews to ensure comprehensive coverage of foundational and emerging literature.

Studies were included if they addressed inflammatory mechanisms or biomarkers associated with breast cancer progression, therapeutic resistance, prognosis, or diagnostic stratification. Priority was given to original research articles, high-quality systematic reviews, and translational studies involving human subjects, with supplementary consideration of relevant preclinical models when they provided mechanistic clarity. Studies focusing exclusively on non-inflammatory genomic markers without immune or inflammatory context were excluded unless directly relevant to integrative biomarker frameworks. Data extraction focused on four principal domains: (i) inflammatory mediators implicated in resistance pathways (e.g., cytokines, chemokines, signaling cascades), (ii) immune cell–based and systemic inflammatory indices, (iii) multi-omics and liquid biopsy–derived inflammatory signatures, and (iv) clinical applications in diagnosis, prognosis, and therapeutic decision-making. Particular attention was given to consistency of findings across breast cancer subtypes, including hormone receptor–positive, HER2-positive, and triple-negative disease.

Evidence was organized thematically to reflect biological mechanisms, biomarker development, and clinical translation. Rather than performing quantitative meta-analysis, emphasis was placed on integrating converging lines of evidence to construct a coherent conceptual framework of inflammation-driven treatment resistance. This approach enabled the identification of recurring patterns, mechanistic overlaps, and translational gaps across heterogeneous study designs. To enhance interpretive rigor, studies were critically appraised for methodological quality, including sample size, assay robustness, statistical validation, and reproducibility of findings. Greater weight was assigned to studies employing longitudinal designs, multi-platform biomarker assessment, or external validation cohorts, given their relevance to clinical applicability. Emerging computational approaches, including machine learning and network-based analyses, were also considered in evaluating the evolving landscape of inflammatory biomarker integration. Finally, the synthesis was contextualized within the broader framework of precision oncology and translational cancer research. This allowed for the identification of current limitations in biomarker standardization, clinical implementation, and cross-platform harmonization, as well as the articulation of future research directions necessary to advance next-generation inflammatory signatures toward routine clinical use.

## 3 Inflammatory mechanisms underlying therapeutic resistance

Therapeutic resistance in breast cancer is increasingly understood as a systems-level phenomenon in which tumor-intrinsic adaptations are tightly coupled to a persistently activated inflammatory microenvironment. Within this context, malignant cells co-opt inflammatory signaling to sustain proliferation, evade cell death, and remodel surrounding stromal and immune compartments. The result is a self-reinforcing ecosystem that attenuates drug efficacy across endocrine, cytotoxic, targeted, and immune-based therapies (16, 17). A central feature of this process is the dysregulated production of pro-inflammatory cytokines and growth factors. Interleukin-6 (IL-6), interleukin-8 (IL-8), tumor necrosis factor-alpha (TNF-α), and transforming growth factor-beta (TGF-β) are consistently implicated as nodal mediators linking inflammation to resistance. IL-6–driven activation of the JAK/STAT3 axis promotes transcriptional programs that enhance tumor cell survival, inhibit apoptosis, and sustain cancer stem–like phenotypes. Similarly, IL-8 signaling through CXCR1/2 facilitates chemotaxis, angiogenesis, and enrichment of stem-like subpopulations that exhibit intrinsic drug tolerance. TNF-α amplifies these effects via canonical NF-κB activation, inducing anti-apoptotic gene expression and reinforcing a pro-survival inflammatory state. TGF-β, while context-dependent, is particularly influential in driving epithelial–mesenchymal transition (EMT), a phenotypic shift associated with invasiveness, immune evasion, and resistance to multiple therapeutic modalities (18, 19).

These cytokine-driven pathways converge on key transcriptional regulators, notably NF-κB and STAT3, which function as master integrators of inflammatory and oncogenic signaling. Persistent activation of these factors establishes positive feedback loops, whereby inflammatory mediators induce their own expression and sustain downstream effector pathways. This signaling persistence not only buffers tumor cells against therapeutic stress but also promotes metabolic reprogramming, oxidative stress tolerance, and epigenetic plasticity—features that collectively enhance adaptive resistance (20, 21). Equally critical is the role of the tumor microenvironment (TME), where immune and stromal cell populations are dynamically reprogrammed under inflammatory cues. Tumor-associated macrophages (TAMs) often adopt an M2-like, immunosuppressive phenotype characterized by secretion of IL-10, TGF-β, and vascular endothelial growth factor (VEGF), thereby promoting angiogenesis and dampening cytotoxic T-cell activity. Myeloid-derived suppressor cells (MDSCs) accumulate in response to tumor-derived factors and inhibit T-cell proliferation through arginase activity, nitric oxide production, and reactive oxygen species generation. Neutrophils, particularly in their tumor-associated (N2-like) state, contribute to extracellular matrix remodeling, metastatic dissemination, and resistance to therapy through the release of proteases and neutrophil extracellular traps (22, 23).

This immunosuppressive milieu directly undermines the efficacy of immunotherapies, including immune checkpoint inhibitors, by restricting T-cell infiltration and function. Moreover, chronic inflammation alters antigen presentation and promotes expression of inhibitory ligands such as PD-L1, further facilitating immune escape. In hormone receptor–positive breast cancer, inflammatory signaling has been shown to crosstalk with estrogen receptor (ER) pathways, leading to ligand-independent activation and reduced sensitivity to endocrine therapies. In HER2-positive disease, inflammatory mediators can activate compensatory signaling pathways that bypass HER2 inhibition, thereby contributing to resistance to agents such as trastuzumab (24, 25). Another important dimension is the contribution of inflammasome activation and innate immune sensing pathways. Activation of complexes such as the NLRP3 inflammasome leads to maturation of interleukin-1β (IL-1β) and interleukin-18 (IL-18), further amplifying local and systemic inflammation. These mediators enhance tumor invasiveness and may promote resistance by modulating both cancer cell behavior and immune cell recruitment (26, 27).

Beyond local effects, systemic inflammation exerts measurable influences on disease trajectory. Elevated peripheral inflammatory indices—such as neutrophil-to-lymphocyte ratio (NLR) and platelet-to-lymphocyte ratio (PLR)—reflect a shift toward a pro-tumorigenic systemic state and correlate with poor therapeutic response. These indices likely mirror underlying biological processes, including myelopoiesis skewing, lymphocyte suppression, and cytokine-driven hematopoietic reprogramming (28, 29). Inflammation-associated resistance is not static but evolves over time under therapeutic pressure. Treatment itself can induce inflammatory responses, including immunogenic cell stress and cytokine release, which paradoxically may select for resistant clones or reinforce protective niches within the TME. This temporal plasticity underscores the need for longitudinal monitoring of inflammatory dynamics rather than reliance on single time-point assessments (Figure 1) (30).

**FIGURE 1:**
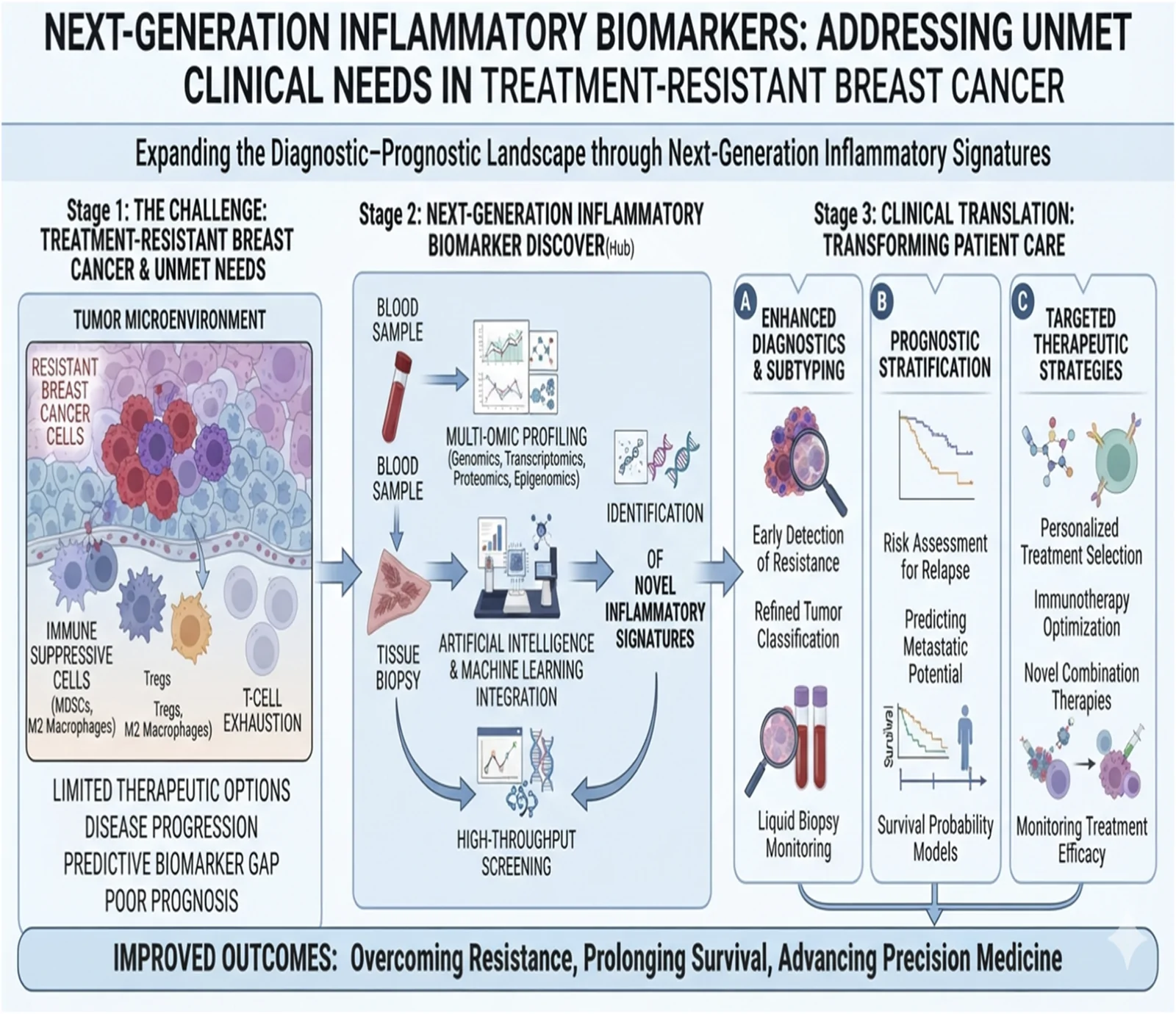
Next-generation inflammatory biomarkers.

## 4 Next-generation inflammatory biomarkers

The evolution of biomarker science in breast cancer is shifting from reductionist, single-analyte approaches toward integrative frameworks that capture the complexity of inflammation as a dynamic, multiscale process. In treatment-resistant disease, this transition is particularly consequential, as inflammatory signaling operates across molecular, cellular, and systemic levels to shape therapeutic response. Next-generation inflammatory biomarkers therefore aim not only to quantify inflammation, but to decode its architecture—linking cytokine networks, immune cell states, and host systemic responses into clinically actionable signatures (31, 32). At the molecular level, multiplexed cytokine and chemokine profiling has provided a more granular view of the inflammatory milieu associated with resistance. Rather than relying on isolated measurements, contemporary platforms quantify coordinated panels of soluble mediators, revealing distinct inflammatory “fingerprints.” Elevated IL-6, IL-8, CXCL10, CCL2, and related chemokines frequently co-occur in resistant phenotypes, reflecting activation of convergent signaling axes such as JAK/STAT and NF-κB. Importantly, the prognostic value of these mediators is amplified when interpreted as networks rather than individual variables, enabling the identification of synergistic interactions and threshold effects that more accurately predict therapeutic failure (33).

Complementing soluble factor profiling, immune cell–based biomarkers have undergone substantial refinement with advances in high-dimensional cytometry and single-cell technologies. Traditional indices such as the neutrophil-to-lymphocyte ratio (NLR), platelet-to-lymphocyte ratio (PLR), and lymphocyte-to-monocyte ratio (LMR) remain clinically accessible proxies of systemic inflammation, but their interpretive power is increasingly enhanced by incorporating phenotypic and functional attributes of constituent cells. For instance, distinguishing between effector and exhausted T-cell subsets, or between pro-inflammatory (M1-like) and immunosuppressive (M2-like) macrophage states, provides deeper insight into immune competence and tumor-promoting inflammation. Within the tumor microenvironment, the density, spatial distribution, and activation status of tumor-infiltrating lymphocytes (TILs) further refine prognostic stratification, particularly in aggressive subtypes such as triple-negative breast cancer (34–36). A major advance in this field is the emergence of multi-omics–derived inflammatory signatures. By integrating transcriptomic, proteomic, epigenomic, and metabolomic data, these approaches capture pathway-level perturbations that are not discernible through single-platform analyses. Inflammation-related gene expression panels, for example, can stratify patients based on activation of interferon signaling, cytokine cascades, or myeloid-driven immunosuppression. Proteomic and metabolomic layers add functional context, reflecting downstream effector activity and metabolic rewiring associated with chronic inflammation. When harmonized through computational modeling, these datasets yield composite signatures with superior predictive accuracy for both treatment response and clinical outcomes (37, 38).

Liquid biopsy technologies have further expanded the scope and clinical feasibility of inflammatory biomarker assessment. Circulating tumor cells (CTCs), cell-free DNA (cfDNA), and extracellular vesicles (EVs) provide minimally invasive windows into tumor biology, and their integration with inflammatory readouts enhances diagnostic resolution. Of particular interest are exosome-associated microRNAs (miRNAs) and proteins that regulate or reflect inflammatory signaling pathways. These circulating factors can be serially measured, enabling real-time tracking of evolving resistance and offering an early indication of therapeutic inefficacy before radiographic progression becomes apparent (39, 40). Another important dimension is the development of composite inflammatory indices that integrate laboratory, molecular, and clinical parameters into unified scoring systems. Such indices move beyond descriptive biomarkers toward decision-support tools capable of guiding treatment selection and monitoring. For instance, algorithms that combine cytokine levels, immune cell ratios, and gene expression profiles can generate individualized risk scores, facilitating precision stratification in both early-stage and metastatic settings (41, 42). Computational advances, particularly in machine learning and network biology, are accelerating the translation of these complex datasets into clinically usable formats. These methods can identify non-linear relationships, latent variables, and predictive patterns within high-dimensional data, thereby uncovering biomarker combinations that would be difficult to discern through conventional statistical approaches. Importantly, these models are increasingly being trained on longitudinal datasets, allowing them to capture temporal dynamics in inflammatory signaling that are critical for understanding the evolution of resistance (Table 1) (43).

**TABLE 1:** Next-generation inflammatory biomarkers in treatment-resistant breast cancer.

Biomarker class	Key biomarkers	Biological role	Potential clinical application
Leukocyte-derived indices	NLR, LMR	Reflect neutrophil/myeloid activation relative to adaptive immunity	Prognostic stratification; treatment-response monitoring
Platelet–immune indices	PLR, SII	Integrate platelet activation, neutrophilic inflammation, and lymphocyte depletion	Risk assessment; metastatic and resistance prediction
Myeloid inflammatory indices	SIRI, AISI	Capture neutrophil, monocyte, platelet, and lymphocyte interactions	Systemic inflammatory profiling; prognostic assessment
Acute-phase proteins	CRP, fibrinogen	Reflect systemic inflammation and inflammation–coagulation coupling	Disease burden and outcome assessment
Cytokine signatures	IL-6, IL-8, IL-1β, TNF-α, TGF-β	Promote tumour survival, angiogenesis, EMT, immune suppression, and resistance	Mechanistic phenotyping; predictive biomarker development
Myeloid-cell signatures	TAMs, MDSCs	Promote immune suppression, angiogenesis, invasion, and tumour persistence	Resistance and immunotherapy-response assessment
Neutrophil/NET biomarkers	cfDNA, citrullinated histone H3, MPO–DNA complexes	Indicate NET formation, immune escape, thrombosis, and metastasis	Metastatic-risk and resistance monitoring
Thromboinflammatory markers	D-dimer, tissue factor, thrombin-related markers	Connect inflammation with coagulation and tumour dissemination	Thrombotic and advanced-disease risk assessment
Tumour-derived liquid biopsy markers	ctDNA, CTCs, extracellular vesicles	Reflect tumour burden, molecular evolution, and emerging resistance	Early molecular response and resistance detection
Integrated multimodal signatures	Inflammatory indices + cytokines + ctDNA + immune/molecular data	Capture dynamic tumour–host interactions	Precision risk prediction and clinical decision support

Abbreviations: AISI, aggregate index of systemic inflammation; CTCs, circulating tumour cells; ctDNA, circulating tumour DNA; LMR, lymphocyte-to-monocyte ratio; MPO, myeloperoxidase; MDSCs, myeloid-derived suppressor cells; NETs, neutrophil extracellular traps; NLR, neutrophil-to-lymphocyte ratio; PLR, platelet-to-lymphocyte ratio; SII, systemic immune-inflammation index; SIRI, systemic inflammation response index; TAMs, tumour-associated macrophages.

## 5 Diagnostic applications

The diagnostic utility of next-generation inflammatory signatures in treatment-resistant breast cancer lies in their capacity to detect biologically meaningful changes that precede overt clinical or radiographic progression. Unlike conventional diagnostics—which are largely anchored in static histopathologic and genomic assessments—these signatures provide a dynamic readout of tumor–host interactions, capturing evolving inflammatory states that signal therapeutic escape. This shift from static to adaptive diagnostics is particularly valuable in refractory disease, where early identification of resistance can substantially influence clinical decision-making (44). One of the most immediate applications is the early detection of emerging resistance during ongoing therapy. Longitudinal profiling of cytokine networks, immune cell distributions, and circulating inflammatory mediators enables the identification of subtle but coordinated shifts indicative of treatment failure. For instance, rising levels of IL-6, IL-8, or chemokine clusters, in conjunction with declining cytotoxic lymphocyte activity, may signal the establishment of a pro-tumorigenic inflammatory milieu even when imaging studies remain stable. Such early warning signals create a window for timely therapeutic modification, potentially preventing full clinical relapse (45).

In parallel, inflammatory signatures can enhance baseline diagnostic stratification by complementing established molecular subtyping frameworks. While hormone receptor status, HER2 expression, and genomic assays define tumor-intrinsic characteristics, they provide limited insight into the immunologic and inflammatory contexture that modulates treatment response. Integrating inflammatory biomarker panels at diagnosis enables a more comprehensive classification, distinguishing patients with immunologically “hot” versus “cold” tumors, or those with high versus low systemic inflammatory burden. This layered diagnostic approach refines risk categorization and supports more informed selection of initial therapeutic strategies (46, 47). Liquid biopsy platforms have been particularly transformative in this domain. The incorporation of circulating inflammatory mediators—alongside circulating tumor DNA (ctDNA), circulating tumor cells (CTCs), and extracellular vesicles—enables minimally invasive, repeatable assessments of disease status. These platforms facilitate real-time monitoring of tumor evolution, capturing both genomic alterations and inflammatory reprogramming. Importantly, changes in circulating inflammatory profiles often precede detectable increases in tumor burden, thereby enhancing diagnostic sensitivity. This is especially relevant in metastatic settings, where tissue biopsies are logistically challenging and may not adequately reflect spatial heterogeneity (47).

Another critical application is the differentiation between true disease progression and treatment-related effects. In clinical practice, distinguishing tumor progression from pseudoprogression or therapy-induced inflammation—particularly in the context of immunotherapy—can be diagnostically challenging. Multiparametric inflammatory signatures provide additional context by characterizing the quality and trajectory of immune activation. For example, a coordinated increase in effector T-cell activity alongside transient inflammatory cytokine elevation may suggest a productive anti-tumor response, whereas a sustained rise in immunosuppressive mediators and myeloid-derived signals may indicate genuine progression (48, 49). Inflammatory biomarkers also hold promise in identifying minimal residual disease (MRD), a state that often precedes clinical relapse. Highly sensitive detection of low-level inflammatory perturbations, when integrated with molecular residual disease assays, can improve the identification of patients at risk of recurrence following definitive therapy. This application is particularly relevant in early-stage breast cancer, where timely intervention at the MRD stage could alter disease trajectory (50).

From a systems perspective, the integration of inflammatory signatures into diagnostic workflows is increasingly supported by computational tools capable of synthesizing complex datasets into clinically interpretable outputs. Machine learning models can assimilate longitudinal biomarker data, imaging findings, and clinical variables to generate predictive scores or alerts, effectively functioning as decision-support systems. These tools not only enhance diagnostic accuracy but also promote consistency and scalability across clinical settings (51). However, translating these advances into routine practice requires careful standardization and validation. Pre-analytical variables, assay heterogeneity, and inter-individual variability in baseline inflammatory states must be addressed to ensure reliability. Moreover, diagnostic thresholds and decision algorithms need to be calibrated within specific clinical contexts, including tumor subtype, stage, and treatment modality (Table 2) (51).

**TABLE 2:** Diagnostic applications of next-generation inflammatory signatures in treatment-resistant breast cancer.

Diagnostic application	Inflammatory signatures	Clinical value
Baseline risk stratification	NLR, PLR, SII, SIRI, CRP	Identifies patients with high systemic inflammatory burden
Early treatment-response assessment	Serial NLR, SII, cytokines	Detects inflammatory changes that may precede clinical progression
Early resistance detection	Rising SII/SIRI, IL-6, IL-8, NET markers	Signals emerging biological treatment resistance
Minimal residual disease assessment	Inflammatory signatures + ctDNA	Combines tumour-derived and host-response signals
Metastatic-risk assessment	NLR, platelet indices, D-dimer, NET markers	Identifies thromboinflammatory and metastatic phenotypes
Immune phenotype characterisation	TAMs, MDSCs, cytokines, lymphocyte profiles	Defines immunosuppressive versus immune-active states
Molecular subtype refinement	Inflammatory signatures + ER/PR/HER2 status	Adds host inflammatory information to tumour classification
Liquid-biopsy integration	ctDNA + cytokines + inflammatory indices	Enables minimally invasive longitudinal monitoring
Multimodal decision support	Inflammatory + molecular + imaging data	Supports dynamic prediction of treatment response and resistance

Abbreviations: CRP, C-reactive protein; ctDNA, circulating tumour DNA; ER, oestrogen receptor; HER2, human epidermal growth factor receptor 2; IL, interleukin; MDSCs, myeloid-derived suppressor cells; NETs, neutrophil extracellular traps; NLR, neutrophil-to-lymphocyte ratio; PLR, platelet-to-lymphocyte ratio; PR, progesterone receptor; SII, systemic immune-inflammation index; SIRI, systemic inflammation response index; TAMs, tumour-associated macrophages.

## 6 Prognostic implications

Next-generation inflammatory signatures are reshaping prognostic assessment in treatment-resistant breast cancer by providing a multidimensional view of disease biology that extends beyond tumor-intrinsic features. Prognosis in this setting is not solely determined by genomic alterations or tumor burden, but by the dynamic interplay between malignant cells and the host immune–inflammatory milieu. As such, inflammatory biomarkers offer a unique lens through which both tumor aggressiveness and systemic host response can be simultaneously evaluated, yielding more nuanced and temporally responsive prognostic stratification (52, 53). A consistent observation across studies is the association between heightened pro-inflammatory signaling and adverse clinical outcomes. Elevated circulating cytokines—particularly IL-6, IL-8, TNF-α, and TGF-β—correlate with reduced overall survival and progression-free survival in patients with refractory disease. These mediators reflect activation of oncogenic pathways that promote proliferation, angiogenesis, and resistance to apoptosis, while also fostering immunosuppressive conditions that blunt anti-tumor immunity. Importantly, their prognostic value is amplified when considered as coordinated networks rather than isolated analytes, underscoring the importance of systems-level interpretation (54, 55, 64).

Systemic inflammatory indices derived from routine hematologic parameters have also demonstrated robust prognostic relevance. Ratios such as the neutrophil-to-lymphocyte ratio (NLR) and platelet-to-lymphocyte ratio (PLR) serve as accessible surrogates for the balance between pro-tumor inflammation and anti-tumor immune surveillance. Elevated NLR or PLR is consistently associated with poorer outcomes, reflecting a shift toward myeloid predominance, lymphocyte suppression, and cytokine-driven hematopoietic reprogramming. When integrated with molecular and clinical variables, these indices contribute to composite prognostic models with improved discriminatory power (56, 57). At the tissue level, the composition and functional orientation of the tumor microenvironment further refine prognostic assessment. High densities of tumor-infiltrating lymphocytes (TILs), particularly activated CD8^+^ T cells, are generally associated with favorable outcomes and improved response to therapy, especially in triple-negative and HER2-positive subtypes. Conversely, enrichment of immunosuppressive populations—including M2-like tumor-associated macrophages and myeloid-derived suppressor cells—portends a more aggressive disease course and diminished therapeutic responsiveness. Spatial organization also matters; the localization of immune cells relative to tumor nests versus stromal compartments can influence prognostic interpretation, a dimension increasingly captured by spatial transcriptomic technologies (58, 59).

Multi-omics–derived inflammatory signatures represent a significant advance in prognostic modeling. By integrating transcriptomic, proteomic, and metabolic data, these signatures capture pathway-level perturbations that reflect the functional state of the tumor–immune interface. Inflammation-related gene expression panels, for example, can stratify patients into risk categories based on activation of interferon signaling, cytokine cascades, or myeloid-driven suppression. These composite signatures consistently outperform single biomarkers in predicting recurrence, metastatic potential, and survival, particularly when validated across independent cohorts (64). An important strength of inflammatory biomarkers is their dynamic nature, which enables longitudinal prognostication. Unlike static baseline predictors, these signatures can be serially measured to track disease evolution and treatment response over time. Changes in cytokine profiles, immune cell ratios, or composite scores during therapy often precede clinical endpoints, providing early prognostic signals of response or failure. This temporal dimension supports adaptive risk stratification, allowing clinicians to recalibrate prognosis as new data emerge throughout the treatment course (60).

Inflammatory signatures also hold value in predicting therapeutic benefit, thereby bridging prognostic and predictive domains. For instance, patients with pre-existing inflammatory activation characterized by interferon-related gene expression or high TIL density may derive greater benefit from immunotherapies, whereas those with dominant myeloid or cytokine-driven suppression may exhibit resistance unless combination strategies are employed. In hormone receptor–positive disease, inflammatory activation has been linked to poorer response to endocrine therapy, further illustrating the prognostic implications of immune–inflammatory crosstalk (61). Despite their promise, the clinical deployment of these biomarkers requires careful consideration of confounding factors that influence systemic inflammation, including infections, comorbid conditions, and treatment-related effects. Standardization of measurement platforms, definition of clinically relevant thresholds, and validation in diverse patient populations remain essential steps for reliable prognostic application.

## 7 Translational and therapeutic perspectives

The maturation of next-generation inflammatory signatures from descriptive biomarkers to clinically actionable tools is redefining therapeutic strategy in treatment-resistant breast cancer. By resolving the structure and dynamics of tumor–immune crosstalk, these signatures provide a rational basis for patient selection, target prioritization, and adaptive treatment design. The translational challenge is twofold: to convert high-dimensional inflammatory data into decision-grade outputs, and to align those outputs with interventions that meaningfully alter the disease trajectory (62, 63). A central implication is the move toward biomarker-guided combination therapy. Inflammatory profiling can identify dominant resistance circuits—such as IL-6/JAK/STAT3 activation, TNF–NF-κB signaling, or TGF-β–driven stromal remodeling—that are not adequately addressed by standard regimens. Targeting these axes alongside endocrine therapy, HER2-directed agents, chemotherapy, or immune checkpoint blockade offers a mechanistically coherent approach to overcoming resistance. For example, tumors exhibiting STAT3-centric cytokine programs may benefit from JAK/STAT pathway inhibition layered onto existing therapy, whereas TGF-β–high phenotypes may require strategies that simultaneously relieve stromal exclusion and restore T-cell infiltration. The key is contextual pairing: matching the inflammatory dependency of the tumor ecosystem with the most appropriate pharmacologic modulators [64, 65].

Inflammatory signatures also refine immunotherapy deployment. Response to checkpoint inhibitors in breast cancer is heterogeneous and often limited by an immunosuppressive microenvironment. Multidimensional readouts—encompassing interferon signaling, T-cell activation states, myeloid burden, and chemokine gradients—enable discrimination between inflamed, immune-excluded, and immune-desert phenotypes. This stratification informs whether checkpoint blockade is likely to be effective as monotherapy or requires priming strategies (e.g., cytotoxic therapy, radiotherapy, or targeted agents) to convert “cold” tumors into “hot” ones. Conversely, signatures dominated by myeloid suppression or cytokine excess can guide incorporation of agents that reprogram macrophages, deplete suppressor cells, or attenuate pathogenic cytokine loops [66, 67]. The temporal dimension of inflammatory biomarkers is particularly valuable for adaptive therapy. Serial measurement via liquid biopsy—integrating cytokines, chemokines, exosomal cargo, and circulating tumor–derived analytes—enables near–real-time tracking of treatment-induced immune remodeling. Early shifts toward pro-tumor inflammatory states can signal impending resistance, supporting pre-emptive modification of therapy rather than reactive changes after radiographic progression. This dynamic monitoring aligns with emerging adaptive trial designs, where treatment is iteratively adjusted based on longitudinal biomarker trajectories [68].

At a systems level, the integration of inflammatory data with genomics, imaging, and clinical variables is increasingly mediated by computational oncology frameworks. Machine learning models can distill complex, nonlinear relationships into risk scores, response probabilities, and treatment recommendations. Network-based approaches further identify key regulatory nodes within inflammatory circuits that are most amenable to therapeutic intervention. Importantly, these models must be interpretable and externally validated to ensure clinical trust and generalizability, particularly across diverse patient populations and care settings [69]. Inflammatory signatures are also informing drug development and trial enrichment. Biomarker-driven eligibility criteria can enrich clinical trials for patients most likely to benefit from specific immunomodulatory or anti-inflammatory strategies, improving signal detection and reducing attrition. Pharmacodynamic endpoints based on inflammatory readouts—such as modulation of cytokine panels or immune cell states—provide early evidence of target engagement and biological activity. In parallel, co-development of companion diagnostics is becoming essential to translate these agents into routine care [70].

Biological confounding—including intercurrent infections, comorbid inflammatory conditions, and treatment-related effects—must be accounted for in assay interpretation and decision thresholds. Additionally, implementation logistics—cost, turnaround time, and integration into clinical workflows—will determine real-world feasibility. Prospective, multi-center validation studies with predefined clinical utility endpoints are essential to establish practice-changing value [69, 70]. These advances invite a reframing of therapeutic goals in refractory breast cancer: from static regimen selection to continuously informed, biomarker-adaptive management. In this paradigm, inflammatory signatures function not merely as correlates of disease state but as operational guides that direct when to intensify, switch, or de-escalate therapy. As evidence accumulates, the convergence of immunobiology, multi-omics, and computational analytics is poised to translate inflammatory insight into durable clinical benefit, advancing precision oncology for patients with treatment-resistant disease.

## 8 Artificial intelligence for predicting immunotherapy response in NSCLC

Immune checkpoint inhibitors have substantially changed the therapeutic landscape of non-small cell lung cancer (NSCLC), particularly through targeting the programmed cell death protein 1 (PD-1)/programmed death-ligand 1 (PD-L1) axis and, in selected settings, cytotoxic T-lymphocyte-associated protein 4 (CTLA-4). However, clinical responses remain heterogeneous, and a considerable proportion of patients experience primary resistance, transient benefit, or acquired resistance. Accurate identification of patients who are most likely to benefit from immunotherapy therefore remains a major unmet clinical need. Artificial intelligence (AI) offers an opportunity to integrate multiple clinical, radiological, pathological, molecular, and immunological variables to generate more comprehensive predictions of treatment response (62). Early AI approaches have explored routinely available clinical and haematological parameters, including age, sex, smoking history, performance status, tumour stage, inflammatory indices, blood-cell counts, and biochemical variables. Although such models may provide useful prognostic or predictive information, their biological specificity for antitumour immunity is often limited. For example, neutrophil-to-lymphocyte ratio and related inflammatory indices may reflect systemic inflammation and have prognostic associations, but they do not directly quantify the complexity of the tumour immune microenvironment. Consequently, a model that predicts immunotherapy response using routine laboratory variables should not automatically be interpreted as an immune-specific biomarker (63).

Radiomics provides an additional dimension by extracting quantitative imaging features that may capture tumour heterogeneity, necrosis, vascularity, spatial architecture, and treatment-related changes that are not readily appreciated by conventional visual assessment. AI-based radiomic models have therefore been investigated for predicting immunotherapy response and survival. Similarly, digital pathology and pathomics can quantify tumour morphology, immune-cell infiltration, stromal characteristics, spatial relationships, and other features associated with the immune microenvironment. These approaches may complement conventional PD-L1 assessment, which, although clinically important, provides only a limited representation of the dynamic and spatially heterogeneous immune landscape (64). The emerging opportunity lies in multimodal AI, in which imaging, pathology, molecular characteristics, clinical variables, and immune-related measurements are integrated within a unified predictive framework. Such models could potentially capture complementary information from different biological levels. For example, imaging may reflect whole-tumour heterogeneity, pathology may characterise cellular and spatial immune features, molecular data may identify genomic determinants of immune sensitivity or resistance, while longitudinal clinical and laboratory data may capture treatment-associated changes. Integration of these domains could provide a more comprehensive representation of immunotherapy responsiveness than any single biomarker [69].

However, important limitations remain. Many current AI models are developed retrospectively using relatively small or single-institution datasets, and reported performance may therefore reflect institution-specific characteristics rather than generalisable biological relationships. Differences in imaging acquisition, pathology preparation and staining, sequencing platforms, patient selection, treatment regimens, and definitions of treatment response can substantially affect model performance. In addition, multimodal datasets frequently contain missing or inconsistently measured variables, creating challenges for model training and validation [70]. Another important consideration is the distinction between prognostic and predictive information. A biomarker associated with longer survival among patients receiving immunotherapy is not necessarily predictive of benefit from immunotherapy. Demonstrating predictive utility requires appropriate comparison of treatment effects across biomarker-defined groups. Future AI studies should therefore incorporate treatment-controlled or comparative designs whenever feasible and should assess whether AI-guided stratification identifies patients who derive differential benefit from immune checkpoint inhibition. The biological interpretability of AI predictions also requires greater attention. Models based primarily on routine clinical or haematological variables may achieve reasonable predictive performance without identifying the underlying immune mechanisms. Future approaches should increasingly incorporate biologically informative variables, including PD-L1 expression, tumour mutational characteristics, tumour-infiltrating lymphocytes, T-cell states, myeloid-cell populations, immune exhaustion markers, inflammatory cytokines, spatial immune architecture, and circulating tumour-derived biomarkers where appropriately validated. Such integration could help transform AI from a statistical prediction tool into a biologically informed precision-oncology platform [67].

## 9 From biomarker discovery to clinical decision support

The transition from inflammatory biomarker discovery to clinical decision support in treatment-resistant breast cancer requires more than demonstrating statistical associations with survival or treatment response. Although markers such as NLR, SII, SIRI, CRP, cytokines, NET-associated products, and myeloid-cell signatures show considerable prognostic promise, their clinical implementation remains constrained by biological heterogeneity, inconsistent thresholds, pre-analytical variation, inter-platform differences, and limited prospective validation. A biomarker must therefore progress through analytical validity, biological validity, clinical validity, clinical utility, and implementation feasibility before it can reliably influence treatment decisions (57). Next-generation inflammatory signatures should be developed using prospective, multicentre, longitudinal cohorts with harmonised blood collection, processing, storage, assay procedures, and predefined analytical thresholds. Importantly, inflammatory measurements should be interpreted in relation to breast cancer subtype, treatment exposure, infection, metabolic status, corticosteroid use, and other potential confounders. Serial measurements may be more informative than isolated values because treatment resistance is a dynamic process in which inflammatory trajectories may change before conventional radiological progression becomes apparent (58). Computational approaches, including machine learning and multi-omic integration, offer opportunities to combine inflammatory indices with ctDNA, tumour genomics, tumour-infiltrating lymphocytes, proteomics, imaging, and clinicopathological characteristics. However, apparently high-performing models derived from retrospective datasets may be affected by overfitting, selection bias, dataset shift, and inadequate external validation. Accordingly, future models should undergo independent validation, calibration assessment, transparent reporting, and prospective evaluation of clinical utility (59).

## 10 Multimodal artificial intelligence and integrated precision oncology in NSCLC

The increasing availability of radiological, pathological, molecular, clinical, laboratory, and longitudinal patient data has created an opportunity to develop multimodal AI systems capable of analysing NSCLC across multiple biological and clinical dimensions. Unlike single-modality models that derive predictions from imaging, histopathology, genomics, or clinical variables independently, multimodal AI seeks to combine complementary information to generate a more comprehensive representation of tumour biology and patient status. This approach is particularly relevant to NSCLC because disease behaviour reflects interactions among tumour genomics, cellular morphology, immune responses, stromal architecture, systemic inflammation, treatment exposure, and host characteristics (58). Radiomics can provide quantitative information regarding tumour phenotype, spatial heterogeneity, texture, vascular characteristics, and treatment-associated changes that may not be fully captured by conventional radiological interpretation. Pathomics can complement these data by characterising cellular morphology, tumour architecture, stromal composition, immune-cell infiltration, and spatial relationships within digitised histological specimens. Molecular datasets, including genomic and transcriptomic profiles, can provide information about driver alterations, pathway activation, tumour heterogeneity, and potential therapeutic vulnerabilities. Clinical and laboratory variables add information concerning disease stage, comorbidities, systemic inflammation, treatment history, and overall patient status. The integration of these domains may therefore improve diagnostic classification, prognostic assessment, treatment selection, and prediction of therapeutic resistance (59).

A major advantage of multimodal AI is its potential to capture biological information that is distributed across different data types. For example, radiological heterogeneity may reflect underlying molecular or pathological diversity, while spatial immune-cell patterns in pathology may provide context for molecular markers associated with immunotherapy response. Similarly, circulating biomarkers may provide longitudinal information that complements the predominantly cross-sectional information obtained from imaging and tissue specimens. Combining these signals could potentially provide greater predictive value than any individual modality. However, multimodal integration introduces substantial methodological challenges. Data generated from different modalities are rarely acquired using identical protocols or at the same time. A CT scan may precede biopsy, molecular testing may be performed on a different tumour specimen, and laboratory measurements may vary throughout treatment. Temporal misalignment can therefore result in models combining biological information that does not represent the same disease state. Furthermore, imaging, pathology, molecular, and clinical datasets often differ in scale, format, resolution, quality, and completeness (64). Data harmonisation is consequently a central requirement for successful multimodal AI. Imaging data may vary according to scanner manufacturer, acquisition protocol, reconstruction algorithm, and preprocessing procedure. Pathological data may be influenced by tissue fixation, staining, section preparation, slide scanning, and image-processing platforms. Molecular data can differ according to sequencing technologies, laboratory platforms, variant-calling pipelines, and quality-control procedures. Clinical data may also vary in terminology, coding practices, documentation, and institutional workflows. Without appropriate harmonisation, AI models may learn technical or institutional differences rather than clinically meaningful biological patterns (60).

Missing data represent another important challenge. Not every patient undergoes comprehensive molecular testing, digital pathology, advanced imaging, or repeated biomarker assessment. Moreover, missingness may be informative rather than random; for example, molecular testing may preferentially be performed in patients with particular clinical characteristics. Simply excluding patients with incomplete multimodal information may reduce sample size and introduce selection bias, whereas inappropriate imputation may create artificial patterns. Future multimodal models should therefore incorporate transparent and validated strategies for missing-data management, including modality-specific imputation where appropriate, missingness-aware architectures, sensitivity analyses, and evaluation of model performance under incomplete-data conditions (61). The manner in which modalities are integrated also influences model performance. Early-fusion approaches combine features from multiple modalities before prediction, whereas late-fusion approaches first generate modality-specific predictions and subsequently combine them. Intermediate or hybrid strategies may allow each modality to retain its distinctive representation while enabling cross-modal interactions. The optimal approach will depend on dataset size, modality availability, computational requirements, and the clinical question being addressed (62).

Interpretability is particularly important for multimodal models because clinicians need to understand which sources of information contribute to a prediction. Attention mechanisms, feature attribution methods, saliency approaches, and modality-specific explanations may help identify clinically relevant patterns. However, computational explainability should not be equated automatically with biological causality. Transparent validation remains necessary to determine whether model-derived features represent genuine disease mechanisms or dataset-specific associations (64). Multimodal AI may be especially valuable for treatment selection and resistance monitoring. An integrated model could combine baseline imaging, histopathology, molecular characteristics, inflammatory markers, and clinical variables to estimate the probability of response to chemotherapy, targeted therapy, immunotherapy, or combination treatment. Serial integration of imaging and circulating biomarkers could further provide dynamic assessment of treatment response and identify emerging resistance before conventional progression becomes evident [65].

## 11 Technical heterogeneity and dataset shift

The clinical performance and generalisability of AI models in NSCLC are strongly influenced by technical heterogeneity and dataset shift. AI algorithms learn patterns from the data used for model development; therefore, differences between development and deployment environments can substantially alter predictive performance. In medical imaging, variations in scanner manufacturers, acquisition parameters, slice thickness, reconstruction algorithms, contrast administration, image resolution, and preprocessing pipelines may modify radiomic features and deep-learning representations. Consequently, a model trained using images generated under one protocol may perform less reliably when applied to images acquired using different equipment or institutional protocols [65]. Similar challenges occur in digital pathology. Differences in tissue fixation, processing, section thickness, staining intensity, slide preparation, scanner characteristics, image magnification, colour profiles, and image compression can introduce batch effects and domain-specific variation. These technical differences may influence the identification of tumour morphology, immune-cell populations, stromal characteristics, and other pathomic features, even when the underlying biological characteristics are similar. Institutional differences in patient demographics, disease prevalence, treatment pathways, referral patterns, laboratory procedures, and clinical documentation can further contribute to dataset shift [66]. Dataset shift may occur when the statistical distribution of the deployment population differs from that of the training population. This can result in reduced discrimination, poor calibration, increased false-positive or false-negative predictions, and loss of clinical utility. Importantly, high performance in an internal test set does not necessarily demonstrate robustness across independent populations. Models may also inadvertently learn institution-specific or acquisition-specific signals rather than biologically meaningful features, creating a risk of hidden confounding and overfitting.

## 12 Future directions

The future of inflammatory biomarker research in treatment-resistant breast cancer should move from isolated prognostic associations toward validated, longitudinal, multimodal signatures capable of informing clinical decisions. Several priorities are particularly important.

### 12.1 Prospective multicentre validation of inflammatory signatures

Future studies should prospectively evaluate combinations of NLR, PLR, SII, SIRI, CRP, cytokines, NET-associated markers, platelet/coagulation parameters, tumour-associated macrophage and myeloid-derived suppressor-cell profiles. These signatures should be tested across different breast cancer subtypes, disease stages, treatment regimens, and demographic populations. Serial sampling before treatment, during therapy, and at suspected progression should be prioritised to determine whether changes in inflammatory state, rather than single measurements, provide earlier evidence of treatment resistance. Independent external validation and predefined thresholds will be essential for reproducibility.

### 12.2 Standardisation and multi-omics integration

Clinical translation requires harmonisation of blood collection, specimen processing, storage, analytical platforms, quality-control procedures, and reporting standards. Future studies should integrate inflammatory signatures with ctDNA, transcriptomics, proteomics, metabolomics, immune-cell phenotyping, tumour-infiltrating lymphocytes, spatial profiling, and imaging. Computational frameworks should incorporate robust feature selection, batch-effect correction, missing-data management, external validation, calibration, and interpretable machine-learning approaches. Importantly, computational complexity should not compromise clinical feasibility.

### 12.3 Integration with established molecular breast cancer classification

Inflammatory signatures should complement rather than replace established ER, PR, HER2, and molecular classifications. Future models should determine whether inflammatory phenotypes provide additional predictive information within specific molecular subgroups, particularly ER-positive/HER2-negative, HER2-positive, and triple-negative breast cancer. Integrating tumour-intrinsic molecular characteristics with host inflammatory state may provide a more comprehensive representation of treatment resistance than either domain alone.

### 12.4 Biomarker-guided clinical trials of anti-inflammatory strategies

The observation that inflammation is associated with treatment resistance does not establish that suppressing inflammation will improve outcomes. Prospective clinical trials are therefore required to test whether targeting specific inflammatory pathways can enhance standard anticancer therapy. Such trials should use biomarker-defined patient selection, serial inflammatory monitoring, appropriate control groups, and clinically meaningful endpoints including pathological response, progression-free survival, overall survival, and patient-reported outcomes. Mechanistically targeted approaches should be prioritised over nonspecific anti-inflammatory treatment.

### 12.5 Development of dynamic resistance-prediction models

Future research should move beyond static biomarker thresholds toward inflammatory trajectories and treatment-response kinetics. A persistent or rapidly increasing inflammatory burden during therapy may provide an earlier indication of biological resistance than a single elevated measurement. Integrating inflammatory trajectories with ctDNA and imaging could enable earlier recognition of minimal residual disease and emerging resistance.

### 12.6 Equity and implementation in resource-limited settings

Low-cost inflammatory indices derived from routine haematology and biochemistry testing may provide an important entry point for precision oncology where advanced genomic testing is unavailable. Future implementation studies should determine whether tiered approaches—beginning with accessible inflammatory indices and escalating to molecular assays when indicated—can provide clinically useful risk stratification while remaining affordable and scalable. Importantly, validation should include populations from Africa and other historically underrepresented regions to avoid developing biomarkers that perform well only in highly selected populations.

### 12.7 From prognostic association to clinical utility

Ultimately, the field must demonstrate that inflammatory signatures do more than correlate with poor outcomes. Future studies should establish whether their incorporation into clinical pathways changes treatment decisions, identifies resistance earlier, reduces exposure to ineffective therapy, improves patient outcomes, and remains cost-effective. Only after analytical validity, clinical validity, predictive performance, external validation, and clinical utility have been demonstrated should next-generation inflammatory signatures be considered mature clinical decision-support tools.

## 13 Limitations and clinical translation of artificial intelligence in NSCLC

Despite the substantial potential of AI to transform the diagnosis, risk stratification, treatment selection, and monitoring of non-small cell lung cancer (NSCLC), important limitations continue to constrain its translation from research settings into routine clinical practice. A major concern is the quality, representativeness, and heterogeneity of the datasets used for model development. Many published studies rely on retrospective, single-centre datasets that may contain limited numbers of patients, incomplete clinical information, class imbalance, and institution-specific acquisition or treatment patterns. Consequently, apparently high model performance may not necessarily translate to populations with different demographic characteristics, disease prevalence, imaging protocols, pathological workflows, or treatment pathways. Technical heterogeneity represents another major challenge. Differences in computed tomography acquisition parameters, scanner manufacturers, reconstruction algorithms, image resolution, contrast protocols, and preprocessing procedures can alter radiomic features and deep-learning representations. Similar variability occurs in digital pathology because of differences in tissue fixation, staining procedures, slide preparation, scanner platforms, magnification, and image processing. Such variations can produce dataset shift, whereby the statistical characteristics of deployment data differ from those of the training dataset. Models that perform well internally may therefore experience reduced discrimination, calibration, or clinical utility when transferred to another institution. Standardisation, harmonisation, robust preprocessing, and external validation are consequently essential.

Feature reproducibility and methodological transparency are equally important. Radiomic and pathomic features may be sensitive to acquisition and segmentation procedures, while complex deep-learning models may generate predictions that are difficult to interpret biologically. Lack of transparency regarding preprocessing, feature selection, hyperparameter optimisation, handling of missing data, and model development can make independent replication difficult. Appropriate reporting standards, reproducible analytical pipelines, explainable AI approaches, and clearly defined validation strategies are therefore necessary to strengthen confidence in reported findings. Another limitation concerns the integration of multimodal data. The future of precision oncology is likely to involve simultaneous analysis of imaging, pathology, molecular profiles, laboratory parameters, clinical characteristics, treatment history, and longitudinal outcomes. However, these data are often generated at different time points, using different platforms and standards, and with variable completeness. Missing data are common and may be non-random, potentially introducing systematic bias. Effective multimodal AI therefore requires robust data harmonisation, interoperable data structures, appropriate missing-data strategies, temporal alignment, and validation across independent datasets.

The predictive performance of AI should also be distinguished from clinical usefulness. A model may demonstrate excellent area under the receiver operating characteristic curve or other statistical performance measures without improving clinical decision-making or patient outcomes. Clinical translation requires assessment of calibration, decision-curve performance, net clinical benefit, workflow compatibility, interpretability, cost-effectiveness, and the potential consequences of incorrect predictions. Importantly, AI should augment rather than automatically replace clinical judgement, particularly when predictions involve invasive procedures, treatment escalation, withdrawal of therapy, or other high-stakes decisions. In immunotherapy, for example, AI models incorporating routine clinical, haematological, inflammatory, imaging, or pathological variables may identify patients with different probabilities of response. However, some of these predictors are indirect surrogates rather than established measures of antitumour immunity. Future models should therefore increasingly integrate validated immune biomarkers, tumour molecular characteristics, spatial immune features, and longitudinal treatment-response information. This distinction is essential to avoid interpreting statistical associations as evidence of specific biological mechanisms. Clinical workflow integration also presents practical challenges. AI systems must be compatible with existing electronic health records, radiology and pathology information systems, laboratory platforms, and multidisciplinary tumour-board processes. For surgical oncology, AI may support preoperative staging, tumour localisation, assessment of resectability, operative planning, intraoperative decision support, and postoperative risk prediction, but implementation should occur within established surgical workflows and under appropriate clinician oversight. Usability, interoperability, training requirements, alert burden, cybersecurity, and responsibility for AI-assisted decisions must be considered alongside algorithmic accuracy.

## 14 Conclusion

Treatment-resistant breast cancer exemplifies the limitations of static diagnostic and prognostic frameworks in the face of a biologically dynamic disease. The growing recognition of inflammation as a central driver of therapeutic resistance has catalyzed the development of next-generation inflammatory signatures that transcend traditional biomarker paradigms. By integrating cytokine networks, immune cell phenotypes, and systemic inflammatory indices with multi-omics and computational analytics, these signatures provide a more comprehensive and temporally responsive understanding of tumor behavior. Across the diagnostic continuum, inflammatory biomarkers enable earlier detection of resistance, more refined baseline stratification, and real-time monitoring of disease evolution. Prognostically, they capture both tumor aggressiveness and host immune competence, offering superior risk discrimination compared to single-parameter models. Importantly, their translational relevance extends into therapeutic decision-making, where they inform biomarker-guided combination strategies, optimize immunotherapy deployment, and support adaptive treatment approaches grounded in longitudinal disease tracking.
